# Guided Full Mouth Implant Rehabilitation in Atrophic Alveolar Ridges Using TTPHIL ALL TILT® Protocol: A Case Report With Three Years Follow-Up

**DOI:** 10.7759/cureus.47368

**Published:** 2023-10-20

**Authors:** Venkat Nag, Manisha Roy, Manikandhan Ramanathan

**Affiliations:** 1 Prosthodontics, Institute for Dental Implantology, Hyderabad, IND; 2 Oral and Maxillofacial Surgery, Meenakshi Ammal Dental College and Hospital, Chennai, IND

**Keywords:** full mouth rebilitation, tilted implants, cad/cam, ttphil all tilt®, immediate function, minimally invasive, guided surgery, virtual implant planning

## Abstract

Computer-assisted implant planning has become a key diagnostic and therapeutic tool in modern dentistry. This case report emphasizes the possibilities in modern implantology combining virtual implant planning, guided surgery with surgical templates, and immediate function. A 75-year-old female presented with maxillary and mandibular dentures and wanted fixed replacement in minimal appointments. Diagnosis, decision-making, and treatment approaches were based on clinical findings and detailed virtual three-dimensional implant planning. Guided implant placement of six implants in each arch using Tall and Tilted Pin Hole Immediate Loading Technique (TTPHIL ALL TILT^®^), and immediate loading with a provisional fixed dental prosthesis (FDP) was performed fulfilling patient’s functional and esthetic demands in a minimally invasive manner. The final computer-assisted design/computer-assisted manufacturing (CAD/CAM) FDP with a titanium framework and ceramic layering was delivered after six months. At the three-year recall, the implant-supported FDP was free of any complications. Uneventful osseointegration of the dental implants and a healthy peri-implant mucosa were observed. Computer-assisted TTPHIL ALL TILT^®^ technique including three-dimensional virtual implant planning, guided surgery, and CAD/CAM fabrication of provisional and final reconstructions allowed for a concise treatment workflow with favorable esthetic and functional outcomes in this maxillary and mandibular full-mouth case without the need of multiple surgeries in a short treatment time.

## Introduction

Dental implants have been increasingly used as a scientifically and clinically validated treatment modality in patients with complete and partial edentulism [1]. Nevertheless, prolonged healing, multiple appointments, and delayed loading associated with conventional implant loading techniques have been posing a challenge to both patients and clinicians alike [2]. Further, anatomical limitations like fine trabecular bone, pneumatisation of the sinus, and risk of inferior alveolar nerve damage can potentially lengthen the treatment plan [3,4]. In recent years, there has been a surge in newer implant systems and digital protocols that can mitigate complex augmentation procedures and deliver predictable results of immediate function and aesthetics in atrophic alveolar ridges [5]. Based on a computerized tomography (CT) scan and a digitized tooth setup, the prosthetic-driven implants can be virtually planned allowing three-dimensional visualization before implant surgery [6]. Transfer of virtually planned implant positions to the real clinical situation is enhanced by a stereolithographic surgical template [7].

One such technique is the Tall and Tilted Pin Hole Immediate Loading Technique (TTPHIL ALL TILT®) where long (16-24 mm) bi-cortical two-piece implants placed at an angle ranging from 30-40 degrees are used for immediate loading of maxillary and mandibular arches in a flap-less, guided manner [8]. The use of long implants increases the bone-implant contact (BIC) surface area which in turn increases the interlocking between bone and implant [9]. This unique system combines the advantages of both bi-cortical and conventional implantology. These implants utilize basal cortical bone for primary stability and eliminate cantilever as well as the need for surgical augmentation and bypass of vital structures. Thereby, this implant distribution aids in giving 14 teeth in each arch that improved chewing efficiency as against 10-12 teeth of the all-on-4 concept. The present case report aims to describe the workflow of rehabilitating an edentulous atrophic ridge clinical case with dental implants using guided surgery and the TTPHIL ALL TILT® technique.

## Case presentation

Diagnosis and treatment planning

**Figure 1:**
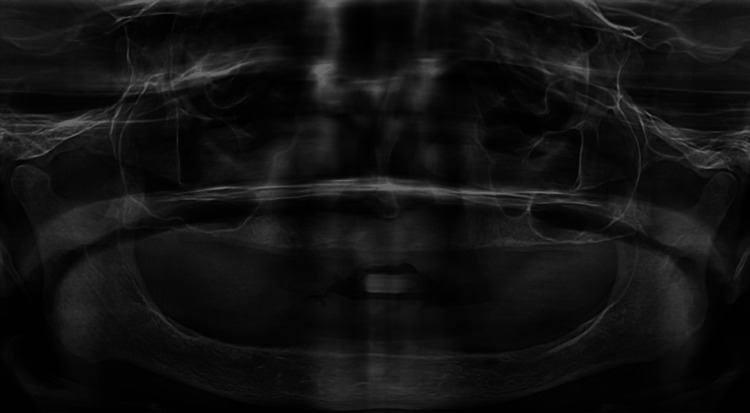
Pre-operative orthopantomogram

A 75-year-old female reported to The Dental Specialists, Hyderabad with a chief complaint of missing teeth in both upper and lower jaw; she had been edentulous for more than 20 years and started wearing complete dentures two to three years back. She wanted the current prosthesis to be replaced by a fixed prosthesis to restore esthetics and speech. Medical history revealed that she was on anticoagulants and thyroxin for five years. The patient did not want to go through an extensive surgical procedure with multiple visits and prolonged treatment time due to its associated morbidity. After a thorough intraoral examination, an orthopantomogram (OPG) (Figure 1) and cone-beam computed tomography (CBCT) scan were taken. The patient was classified as having class C edentulous maxilla and mandible [8]. Diagnostic impressions for both arches were made using alginate impression and a study model was fabricated. Considering her age, medical conditions (ASA type 3), and the amount of residual bone, TTPHIL ALL TILT® Technique for maxillary and mandibular fixed implant rehabilitation was pertinent to provide minimally invasive treatment in minimal appointments. The virtual surgical treatment plan consisted of upper and lower arch digital planning in software and fabrication of the prototyped surgical guide printed on a 3D printer.

Fabrication of stereolithographic models and surgical guides

**Figure 2:**
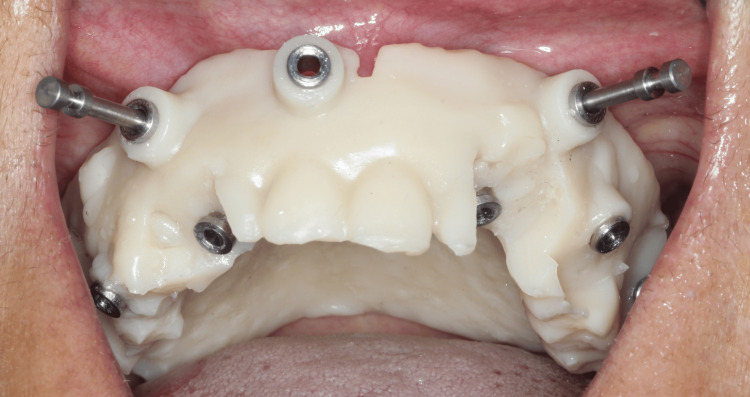
Maxillary surgical guide

**Figure 3:**
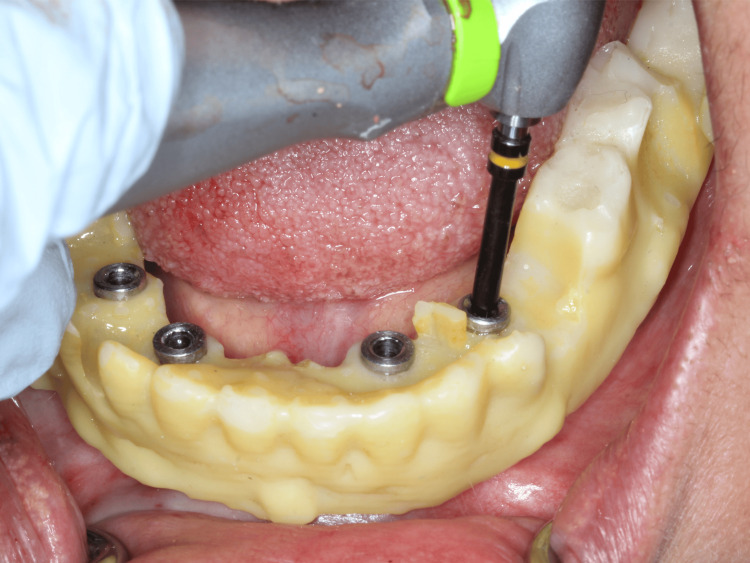
Mandibular surgical guide

Stereolithographic models were made using CBCT DICOM data. Maxillary and mandibular surgical guides were fabricated using Dual Scan technique. Trial dentures were fabricated on diagnostic casts after registering jaw relation. Wash impression with addition silicone light body was made on trial dentures. Five radio-opaque (composite) markers on buccal flange and three on the palatal aspects of trial dentures were placed. Patient was asked to wear the trial denture with markers and a CBCT was taken with the dentures in occlusion ensuring that it was seated properly. Next a CBCT of only the trial dentures with the markers and the wash still in place was taken. Both scans were overlapped and implant sites were planned in a digital implant planning software. After finalizing the planning and the design of the surgical guide, the DICOM data files were sent to a milling lab for the fabrication of prototyped stereolithographic maxillary and mandibular surgical guides using a 3D printer (Figures 2, 3).

Surgical phase

During the day of surgery, a single dose 2 g of oral amoxicillin and clavulanic acid was administered one hour before the commencement of surgery. This treatment continued for five days (1 g amoxicillin and clavulanic acid twice a day). Prior to the start of surgery, the patient rinsed with 0.2% chlorhexidine for 1 min. Local crestal anesthesia and sub periosteal infiltration was administered using a 2% lignocaine solution with epinephrine 1:200,000 through guide sleeves.

Maxilla

**Figure 4:**
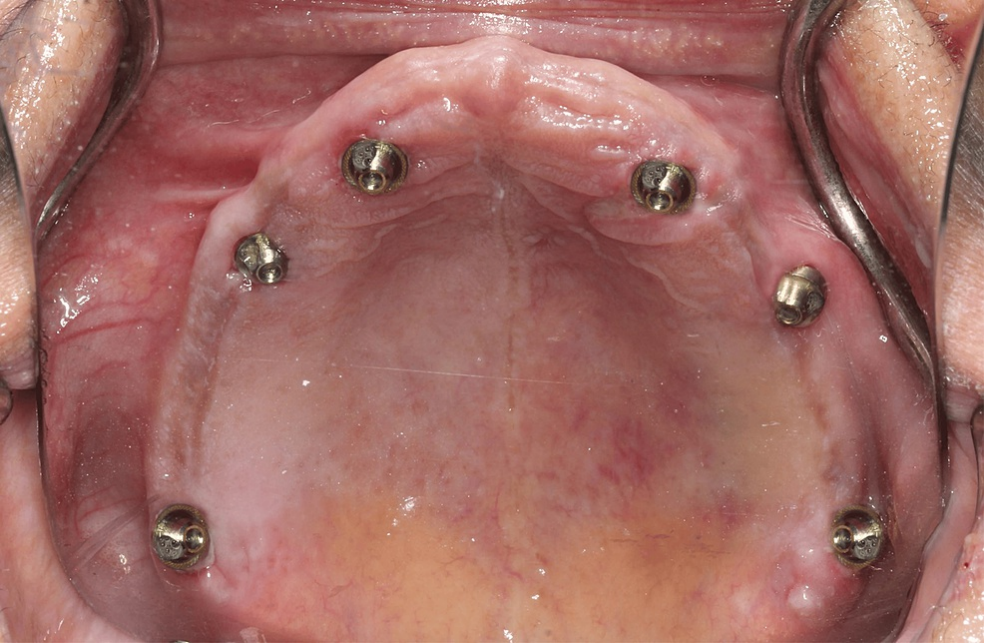
Multi-unit abutments placed over the dental implants in mandibular arch

Surgical guide was placed on the upper jaw using anchor pins. A lancet drill (1.4 mm) was placed through the guide hole of the surgical guide in the second premolar region up to a depth of 6 mm that acts as a guidance path for implant placement. RVG in periapical view was captured to confirm that sinus was bypassed anterior to the anterior extension of maxillary sinus wall. Next, the guide is removed, and a proper osteotomy is created using a single drill (1.8 mm), in the direction of the path created by the lancet drill. This drill was able to reach through and through to the nasal cortical bone and a cortical dip of the drill was felt. A 3.75 x 11.5 mm implant (Bioline I, BiolineDental GmbH&Co.KG) was placed in the osteotomy site with a torque of 60 Ncm. This same procedure to create osteotomies for all the other five sites was followed. The second area of osteotomy was performed in the region of canine, 3.75 x 16 mm implant was placed. The third implant in pterygoid region (3.75 x 16 mm) was placed with the implant tip directed superiorly, distally and palatally, approximately 30 degrees to occlusal plane, engaging the pterygoid cortex posteriorly. The same guided implant placement technique was followed on the contralateral side. All implants were torqued to 60 Ncm. Multi-unit abutments were placed for canine (15 degrees), second premolar (30 degrees), second molar pterygoid region (45 degrees) such that the multi-unit handles parallel each other and in turn would also compensate the implant angulation (Figure 4).

Mandible

**Figure 5:**
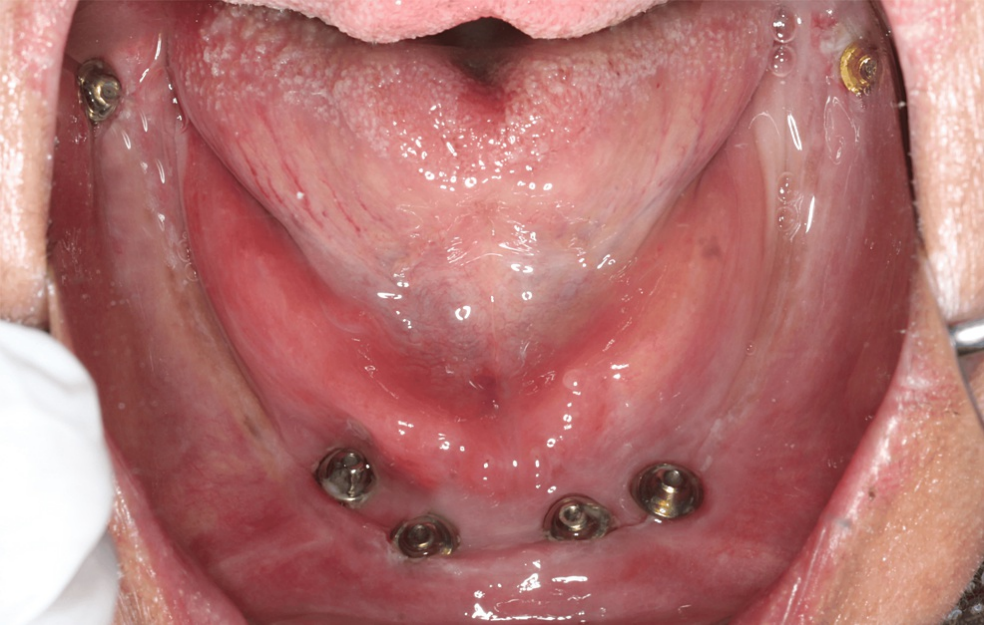
Multi-unit abutments placed over the dental implants in mandibular arch

Using stereolithographic surgical guide for the mandible the same drilling method was used as maxilla. The first implant (3.75 x 13 mm) was placed in the region of second premolar and 2 mm anterior to mental nerve loop. The second implant (3.75 x 13 mm) was placed in the region of canine and third implant (3.75 x 11.5 mm) was placed posteriorly in the region of second molars with entry point being lingual and implant tip exiting buccally. All implants were torqued to 60 Ncm. Multi-unit abutments were placed for canine (20 degrees), second premolar (30 degrees), second molar pterygoid region (50 degrees). The same sequence was repeated on the contralateral side (Figure 5). Postoperative CBCT confirmed the position implants were correctly positioned, maintaining a safe distance from the maxillary sinus and nerve.

Prosthetic phase

**Figure 6:**
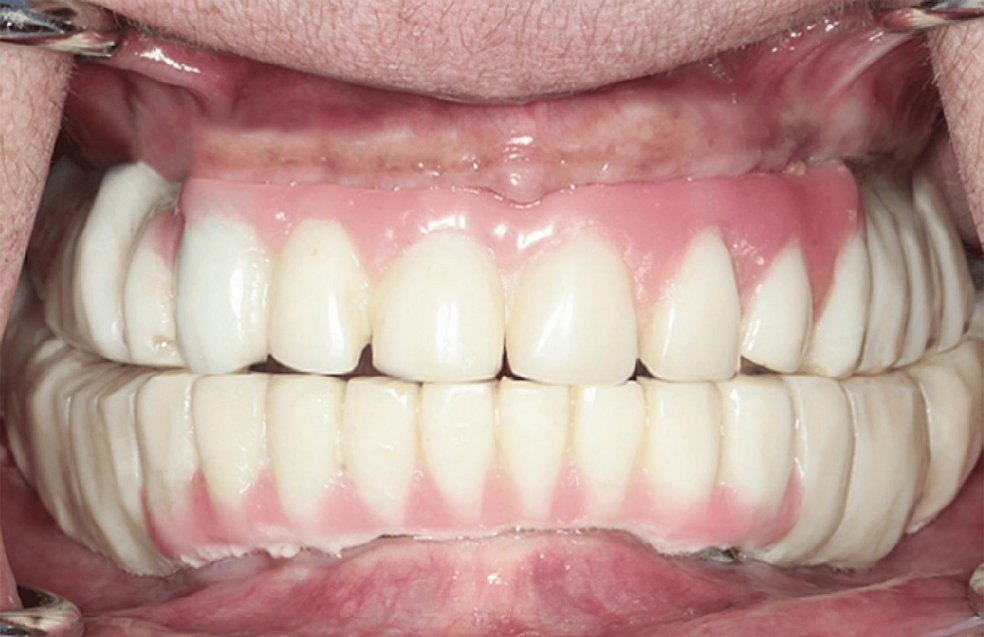
Implant supported provisional fixed dental prosthesis

**Figure 7:**
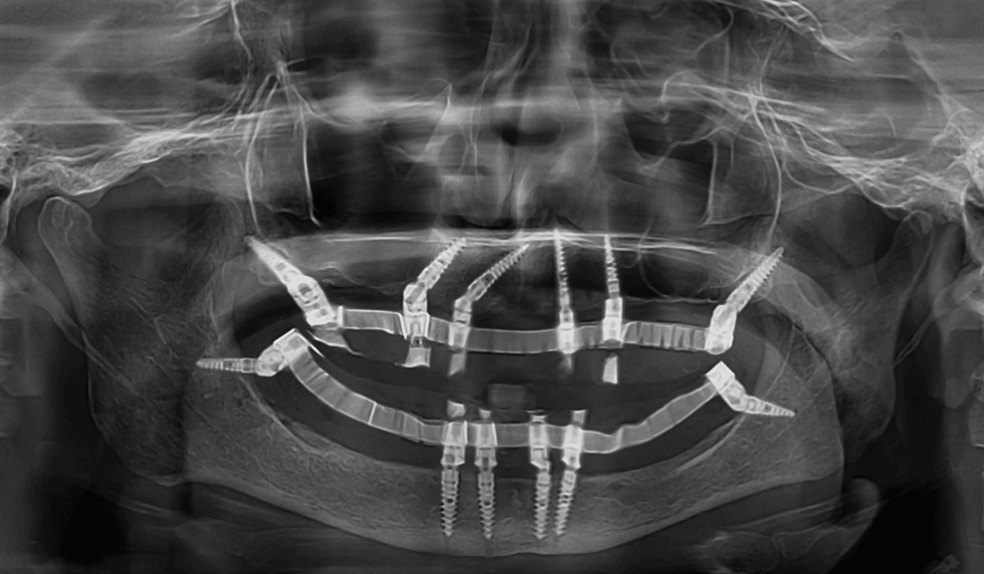
Orthopantomogram of the patient after provisional prosthesis placement

Multiunit impression copings were attached to abutments and splinted using wire-bars and pattern resin to ensure an accurate transfer of implant positions. An open tray poly-vinyl siloxane impression of both arches was made to detect the position of implants and soft tissue. Implant analogs were attached to copings in master impression, master casts were obtained. Titanium-based metal cylinders (Ti-bases) were screwed onto the multiunit abutments and Aluwax (Maarc perfect bite, Hyvincare, India) was engaged around metal bases to record jaw relations. The jaw relation record was articulated, and titanium cylinders attached to each implant analogs of both master casts. Titanium metal sheets 1.5 mm thick was welded around each titanium cylinders using an in-house welding machine to splint the cylinders. A provisional maxillary and mandibular prosthesis were fabricated with high, and the metal framework created in the previous steps. The provisional prosthesis was delivered to the patient on the next day after checking occlusion (Figures 6, 7). The general condition was satisfactory in the next follow-ups; the postoperative region was without any exaggerated inflammatory changes.

**Figure 8:**
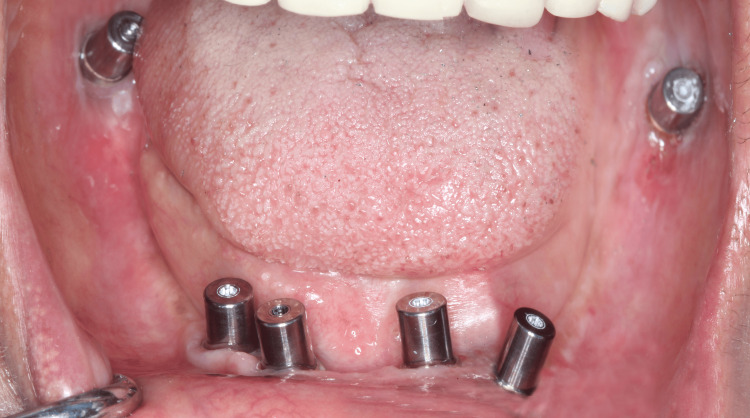
Scan bodies attached to multi-units for digital impressions

**Figure 9:**
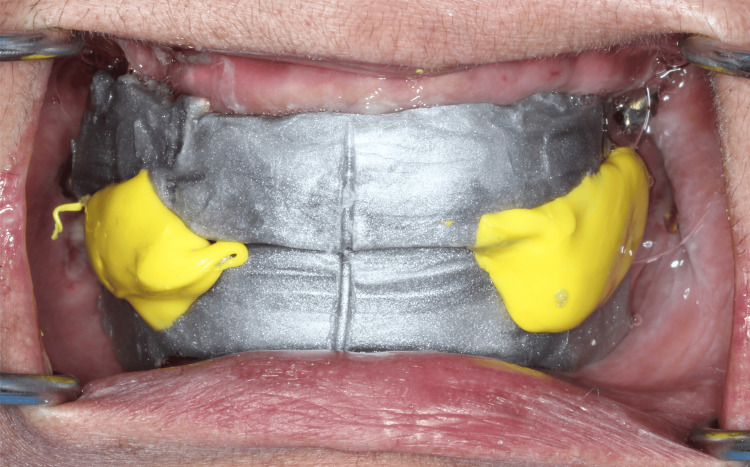
Jaw relation record

**Figure 10:**
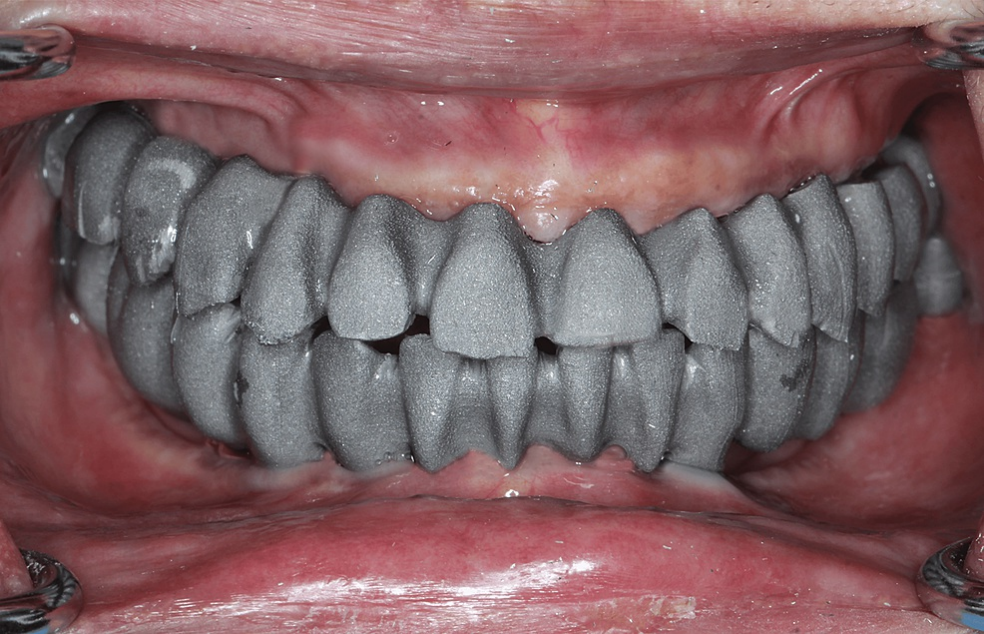
Try-in of metal framework

**Figure 11:**
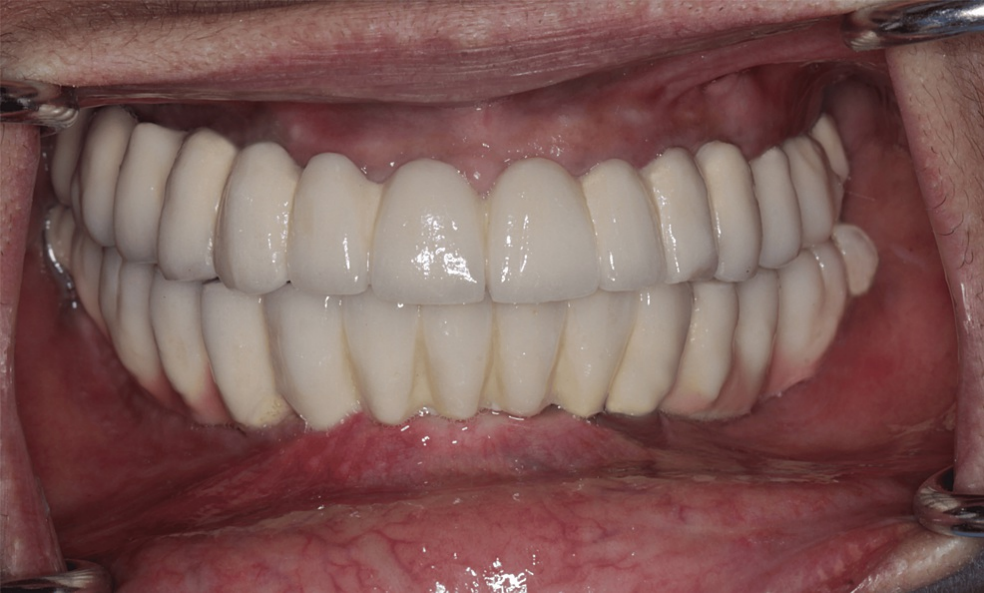
Final implant supported screw retained fixed dental prosthesis

After the provisional phase of four months, a second digital maxillary and mandibular impressions were made with intra-oral scanner using scan bodies that were attached to the multi-units (Figure 8). After sealing the hole of scan bodies, putty and light body dual impressions were made. Following digital impressions, verification jigs were tried in the patient's mouth to confirm implant positions. Jaw relation records were taken (Figure 9) and computer-assisted design/computer-assisted manufacturing (CAD/CAM) Co-Cr metal frameworks were fabricated for both maxillary and mandibular arches. Try in of metal framework was done on the next day to check the fit and seating of screws (Figure 10). The bite was recorded using aluwax and the midline was confirmed. Bisque try-in was done on the same day; esthetics and occlusion in centric and eccentric movements were checked and sent to the lab for glazing. The final prosthesis was screwed onto the multi-unit abutments and occlusion reconfirmed (Figure 11).

**Figure 12:**
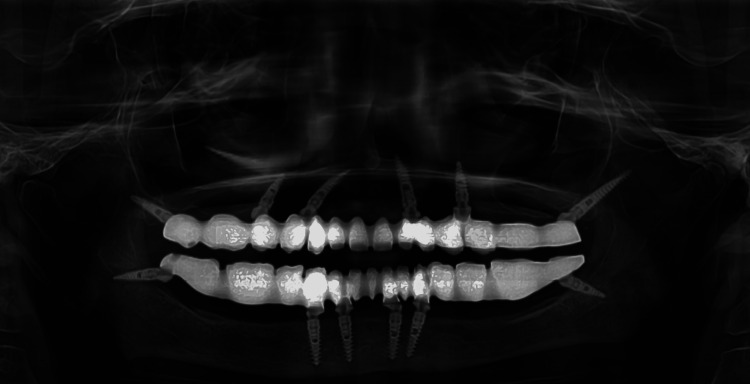
Orthopantomogram of the patient after prosthesis placement

**Figure 13:**
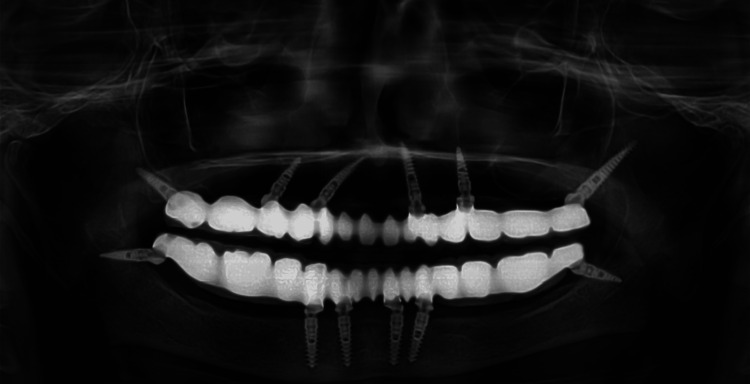
Orthopantomogram of patient at three-year follow-up

Post final prosthesis placement OPG was taken to confirm the fit of the screws and prosthesis (Figure 12). Post prosthesis delivery, instructions were given to maintain proper oral hygiene and good maintenance of the prosthesis. The patient was regularly followed up every three months up to a year and after that every year up to three years. OPG was taken at each follow up visit. The OPG at three-year-follow up revealed a good bone healing and no sign of bone resorption around implants (Figure 13).

## Discussion

The age and medical conditions of the patient (ASA type 3) necessitated the need for minimally invasive implant surgery for fixed full-mouth rehabilitation. TTPHIL ALL TILT® technique allows reduced costs, morbidity rates, and treatment times, as bone augmentation procedures are not needed. Tilted implants permit the placement of longer fixations in the residual bone, which increases the surface area of bone-implant contact and the primary stability by providing anchorage to more than one cortical plate [9]. Other benefits include the elimination of cantilever length and stress reduction in both cancellous and cortical bone; and better distribution of the occlusal forces [9]. This tilted implant configuration prevented cantilever extensions and provided better anterior-posterior stress distribution that prevents angulated torque moments on the implants, frequently resulting in resorption in the cortical bone around the distal implant neck [10]. Digital planning has been used for greater predictability and precision in the planning of dental implants, giving greater chances of successful surgery along with the technique performed correctly [11]. Using 3D planning software, the ideal implant size and location in the alveolar bone were designed according to the position and contour of the desired restoration. Guided surgery has been done as it is proven to be a reliable and precise method that reduces the damage to the alveolar nerve, sinus perforation, and fenestration [12]. Flap reflection causes cessation of blood supply from the soft tissue to the bone, thus leaving poorly vascularized cortical bone promoting bone resorption in the healing phase [13]. This guide enabled not only precise implant placement but also flapless surgery whose advantage is the minimal surgical procedure that supports the preservation of the blood circulation in the soft tissues, which may affect the soft-tissue architecture and reduce bone loss [14]. Further, post-operative swelling, pain, and discomfort are greatly reduced with no need for sutures [13].

Subcrestal placement decreases the risk of exposure to the metal top of the implant creates a harmoniously esthetic emergence profile and improves soft tissue esthetic results [15]. Single drill decreases time and heat to the bone tissue and enables under preparation of the implant bedsite, thus, increasing implant stability [16]. Single-stage surgery reduces the horizontal bone remodeling around the immediately restored, subcrustal-placed implants [17]. The use of healing abutments contributes to the preservation of marginal bone and the establishment of a more favorable peri-implant biological width. Baggi, et al. have found that the platform switching concept along with subcrestal positioning demonstrated better stress distribution in bone and a lower risk of bone overload [18]. The rigidly splinted screw-retained metal-ceramic prosthesis was delivered as they have shown to be superior to the cement-retained prosthesis, due to the advantages of retrievability and prevention of cement-induced peri-implantitis. With increased occlusal units, there is increased chewing efficiency as the number of chewing strokes required before swallowing is significantly reduced [19].

With all the advantages of the concepts mentioned, the TTPHIL ALL TILT® technique for maxillary and mandibular rehabilitation has provided the patient with a minimally invasive, digitalized, and predictable fixed implant treatment option with better chewing efficiency and immediate function. Encompassing the advantages of efficient workflow using computer-assisted implantology, this technique enabled the clinician to provide a quick, immediate, and non-grafting solution which aided in longevity by eliminating the cantilever. The clinical and radiographic assessment of the patient during the three-year follow-up period demonstrated that the technique was successful in a 75-year-old patient with atrophic ridge. This case report intends to contribute to the limited literature available on the TTPHIL ALL TILT® technique which is a comparatively new technique in the field of tilted implantology-related reports and research. Nevertheless, long-term follow-up, the use of the technique in more patients, and case-control studies will help validate the efficiency of the technique.

## Conclusions

The present case report explained in detail the workflow and favorable treatment outcome achieved using computer-assisted implantology in a 75-year-old patient. Atrophic ridge rehabilitation, which is usually a challenging clinical case for implant clinicians, was successfully completed as inferred from the three-year follow-up of the patient. The fabrication of a surgical guide, an immediate provisional fixed dental prosthesis, and subsequently, the final CAD/CAM framework reconstruction has facilitated fulfilling the patient’s wish for immediate function in a minimally invasive manner using the TTPHIL-ALL-TILT® protocol.
